# DdaCrz1, a C_2_H_2_-Type Transcription Factor, Regulates Growth, Conidiation, and Stress Resistance in the Nematode-Trapping Fungus *Drechslerella dactyloides*

**DOI:** 10.3390/jof8070750

**Published:** 2022-07-20

**Authors:** Xiaozhou Zhao, Yani Fan, Meichun Xiang, Seogchan Kang, Shunxian Wang, Xingzhong Liu

**Affiliations:** 1 State Key Laboratory of Medicinal Chemical Biology, Key Laboratory of Molecular Microbiology and Technology of the Ministry of Education, Department of Microbiology, College of Life Science, Nankai University, Tianjin 300071, China; 1120190488@mail.nankai.edu.cn; 2 State Key Laboratory of Mycology, Institute of Microbiology, Chinese Academy of Sciences, Beijing 100101, China; hnfyn1234@163.com (Y.F.); xiangmc@im.ac.cn (M.X.); 3 University of Chinese Academy of Sciences, Beijing 100049, China; 4 Department of Plant Pathology & Environmental Microbiology, The Pennsylvania State University, Texas, PA 16802, USA; sxk55@psu.edu

**Keywords:** calcium signaling, *Drechslerella dactyloides*, *DdaCrz1*, fungal growth, predatism, stress response

## Abstract

The Ca^2+^/calmodulin-dependent signaling pathway regulates diverse cellular processes. Calcineurin is a calcium-dependent phosphatase acting in fungi mainly through Crz1, a zinc finger transcription factor. Although the likely involvement of Ca^2+^ in fungal carnivorism has been documented, how *Crz1* functions in nematode-trapping fungi remains unknown. Here, we identified the *Crz1* gene (named as *DdaCrz1*) in *Drechslerella dactyloides,* a species that forms constricting rings to trap nematodes. The deletion of *DdaCrz1* significantly reduced hyphal growth and conidiation, trap formation, and ring cell inflation. Moreover, the mutation increased sensitivity to Mn^2+^ but decreased sensitivity to Ca^2+^, Mg^2+^, Zn^2+^, and Li^+^. Similarly, the mutant showed increased tolerance to osmotic stress but was more sensitive to Congo red, a cell wall-damaging agent. Our results confirmed the critical roles of the Ca^2+^/calmodulin-dependent signaling pathway in regulating growth, conidiation, and the stress response, and suggested its involvement in trapping nematodes.

## 1. Introduction

Calcineurin, the Ca^2+^/calmodulin-dependent serine-threonine-specific protein phosphatase that is highly conserved among single-cell and multicellular eukaryotes, is involved in regulating diverse processes [1,2,3,4,5]. The Ca^2+^-dependent signaling pathway is triggered by receptors that perceive external stimuli, resulting in Ca^2+^ binding to calmodulin, which subsequently activates calcineurin [6,7]. Calcineurin is composed of a catalytic subunit A (CNA) and a regulatory subunit B (CNB) [8,9]. The expression of the genes downstream of calcineurin is mainly activated by the transcription factor calcineurin-responsive zinc finger 1 (Crz1). As the primary mediator of calcineurin signal transduction, Crz1 is dephosphorylated after calcineurin activation and transferred from the cytoplasm to the nucleus, where it binds to calcineurin-dependent response elements (CDREs) in the promoters of target genes [10,11]. *Crz1* is involved in regulating a variety of cellular processes, including cell wall integrity, ion transport, redox processes, carbohydrate metabolism, protein phosphorylation, and vesicle-mediated transport. The *Crz1* in yeasts and filamentous fungi has been extensively characterized. In *Saccharomyces cerevisiae*, *Crz1* regulates the expression of *FKS2*, *PMC1*, *PMR1*, *ENA1*, *GPX2*, and *RCN1*, which are involved in maintaining the cell wall integrity, ion transport, and glucose metabolism [10,12]. In *Candida albicans*, the deletion of *Crz1* caused hypersensitivity to alkaline cations, detergent SDS, and fungicide azoles [13,14,15], and impacted cation homeostasis and virulence [16]. In *Botrytis cinerea*, a filamentous fungus that infects diverse plants, the *Crz1* gene is involved in hyphal morphology, conidiation, sclerotia formation, and host penetration [17]. The knockout of *CrzA* in *Aspergillus fumigatus* resulted in decreased conidiation and virulence and increased sensitivity to ion stress [18]. The deletion of *Crz1* in *Magnaporthe oryzae* impaired its growth in the presence of Ca^2+^ or cell wall degradation agents and reduced conidiation and virulence [19]. These studies underscored the significance of *Crz1* for growth, conidiation, virulence, and stress defense in fungi. 

Nematode-trapping fungi (NTF), which consume nematodes as nutrient sources, influence nematode dynamics in nature. The hyphae of NTF can develop diverse trapping devices, including adhesive networks, adhesive knobs, adhesive columns, and constricting rings [20,21], which can capture vermiform nematodes as nutrient resources [22,23]. Calcium ion is a ubiquitous second messenger in cells. The Ca^2+^/calmodulin-dependent protein kinases (CaMKs) [24] and the low-affinity calcium channel protein [25] encoded by *Arthrobotrys oligospora*, an NTF, have been shown to be involved in hyphae growth, conidiation, trap formation, and stress management. Chen et al. [26] proposed that nematodes activate the G-proteins in the ring cells of *Drechslerella dactyloides*, a model for constricting ring-forming NTF. This activation increases cytoplasmic Ca^2+^, causing the activation of calmodulin and the subsequent opening of water channels for the inflation of constricting rings. These studies indicated that the Ca^2+^ signaling pathway plays crucial roles in NTF.

Our preliminary investigation showed that FK506, a calcineurin inhibitor, could significantly inhibit the growth and trap formation of *D. dactyloides*, suggesting the involvement of *Crz1* in the growth and predatory process. Although a CaMK gene of *D. dactyloides* was cloned and characterized in 2002 [27], its role in Ca^2+^/calmodulin-mediated signaling remains unknown. We identified *DdaCrz1*, a *Crz1* homolog, in *D. dactyloides* and investigated how it affects the growth, conidiation, trap formation, and stress resistance via gene deletion.

## 2. Materials and Methods

### 2.1. Strains and Growth Conditions

The *D. dactyloides* strain 29 (CGMCC3.20198) was isolated from the soil of Motuo, Tibet, China. This strain and its mutants were cultivated on potato dextrose agar (PDA, BDTM, New York, NY, USA), tryptone glucose (TG, BDTM), and corn meal agar (CMA, BDTM) supplemented with 2 g/L KH_2_PO_4_ [28] at 28 ℃. *Caenorhabditis elegans* was maintained on nematode growth medium (NGM) agar plates at 23 ℃ and fed with *Escherichia coli* OP50 strain [29].

### 2.2. Sequence and Phylogenetic Analysis of DdaCrz1

The Crz1 transcription factor DdaCrz1 encoded by *D. dactyloides* was identified via BLASTP using the amino acid sequences of Crz1 encoded by *Aspergillus fumigatus*, *Botrytis cinerea*, and *Schizosaccharomyces pombe* as queries for searching in the NCBI database. The isoelectric points (pI) and molecular weights were predicted using the online software ExPASy-ProtParam tool (https://web.expasy.org/protparam/, accessed on 1 February 2022), and conserved functional domains were identified using SMART (http://smart.embl-heidelberg.de/, accessed on 1 December 2021) and InterProScan (http://www.ebi.ac.uk/interpro/, accessed on 1 December 2021)). Additional Crz1 orthologs encoded by other fungi were retrieved from the NCBI database using BLASTP. A phylogenetic tree based on amino acid sequences of the Crz1 proteins encoded by diverse fungi was constructed using the MEGA7 software package.

### 2.3. Gene Knockout of DdaCrz1

The *DdaCrz1* gene was deleted using a homologous recombination-mediated strategy. The 5′ (2992 bp) and 3′ (3011 bp) (Figure S1) flanking regions of the *DdaCrz1* open reading frame (ORF) were cloned into the upstream and downstream of the hygromycin B resistance gene cassette on the pAg1-H3 vector using the ClonExpress^®^ II One Step Cloning Kit C112 (Vazyme, Nanjing, China) [30,31]. The 5′ flanking region was amplified using primers Crz1-5F and Crz1-5R, and the 3′ flanking region was amplified using primers Crz1-3F and Crz1-3R (Table S1). Subsequently, the resulting plasmid was transformed into *Agrobacterium tumefaciens* strain AGL-1 (Biomed, Beijing, China). *DdaCrz1* was deleted via an improved *A. tumefaciens*-mediated transformation (ATMT) method [31,32]. The AGL-1 transformant containing the plasmid was co-cultured with *D. dactyloides* conidia (ca. 10^6^) harvested from 14-day-old culture on CMA for 7 days. Subsequently, the co-cultures were covered with PDA containing 100 μg/mL hygromycin B (Leagene, Beijing, China) and 400 μg/mL cefotaxime sodium (Solarbio, Beijing, China) for selecting transformants. The resulting transformants were maintained on PDA plates containing 100 μg/mL hygromycin B. Genomic DNA was extracted from the transformants using cetyltrimethylammonium bromide (CTAB) as previously described [33]. Individual transformants were analyzed for the existence of the hygromycin B resistance gene and the deletion of the *DdaCrz1* gene using PCR [34] with two pairs of primers, HYG-540F and HYG-540R, and Crz1-667F and Crz1-667R (Table S1). Confirmed mutants were analyzed via quantitative real-time PCR (qPCR) to check the lack of transcripts. Cultures derived from single conidia were transferred onto PDA plates containing 100 μg/mL hygromycin B for 5 generations [34] to ensure the stability of the mutants before phenotypic characterization.

### 2.4. Complementation of the DdaCrz1 Mutant

To complement the mutation created by *DdaCrz1* deletion, a copy of *DdaCrz1* was introduced into Δ*DdaCrz1-27* via ATMT [32]. The complementation plasmid was constructed using the ClonExpress^®^ II One Step Cloning Kit C112 (Vazyme, Nanjing, China). The *DdaCrz1* gene, including the 5′ (2079 bp) and 3′ (2032 bp) flanking regions, was amplified using primers Re-Crz1-F and Re-Crz1-R (Table S1). Subsequently, the amplified fragment was cloned into a modified pAg1-H3 vector [31] with the G418 resistance gene using one-step cloning. After ATMT, transformants were selected on PDA containing 200 μg/mL G418 (Leagene, Beijing, China). The resulting transformants were screened by PCR using two pairs of primers, G418-617F/G418-617R and Re-Crz1-F/Re-Crz1-R. Subsequently, the transformants containing the introduced copy of *DdaCrz1* were transferred onto PDA plates supplemented with 200 μg/mL G418 for at least 5 generations [34].

### 2.5. Comparison of Mycelial Growth and Conidial Production

The growth rates of strain 29 and its mutants under different nutritional conditions were compared by inoculating 5 mm-diameter discs punched from the edges of 14-day-old CMA culture onto PDA, TG, and CMA plates. During 12 days of incubation at 28 °C, their colony diameters were measured every two days. Conidia from the 14-day-old CMA cultures were harvested by water containing 0.1% Tween-20. The number of conidia produced by each strain was counted using a hemocytometer.

### 2.6. Analyses of Mycelial Growth under Different Stresses

Discs (5 mm in diameter) punched from the edges of 14-day-old cultures of strain 29 and its mutants on CMA plates were placed on the center of individual plates supplemented with various agents stressing fungi. In the initial experiment, the following agents were tested: NaCl and KCl at 0.1 M, 0.2 M, and 0.3 M; sorbitol at 0.2 M, 0.5 M, and 0.8 M; Congo red at 0.1 mg/mL and 0.2 mg/mL; SDS at 0.01% and 0.03%; and menadione at 0.03 mM and 0.06 mM. Subsequently, PDA amended with the following agents was used: 0.03 mM menadione, 0.2 mg/mL Congo red, 0.01% SDS, 0.2 M NaCl, 0.2 M KCl, 0.5 M sorbitol, 0.2 M CaCl_2_, 0.2 M MgCl_2_, 0.2 M LiCl, 0.05 mM ZnCl_2_, and 0.06 mM MnCl_2_. Colony diameters were measured after 14 days (except for 0.06 mM MnCl_2_, which was measured after 25 days) of incubation under 28 °C. For oxidative stress, we chose menadione instead of H_2_O_2_ because *D. dactyloides* is extremely sensitive to H_2_O_2_ and could not grow under the tested concentrations (0.005%, 0.01%, and 0.02%). Relative growth inhibition (RGI) caused by each chemical stress was calculated using the equation (*Dc* − *Dt*)/(*Dc* − *d*) × 100%, where *Dc* and *Dt* refer to the diameters of the unstressed (control) and stressed colonies, respectively, and *d* is the diameter of the inoculated discs [35,36].

### 2.7. Constricting Ring Formation and Inflation after Introducing Nematodes

To compare the ability to form traps, ca. 1000 conidia harvested from strain 29 and its mutants were inoculated onto 2% WA plates and incubated at 28 °C for 3 days. Each plate was supplemented with approximately 1000 *C. elegans*. At 16 h and 24 h after the induction, the numbers of constricting rings and inflated constricting rings within a 4 cm^2^ area of each colony were counted.

### 2.8. RNA Extraction

Conidia of strain 29 and its mutants were plated onto WA plates (about 10^5^ per plate) overlaid with a cellophane membrane [37]. Mycelia above the membrane were harvested after 3 days of incubation, firstly before adding nematodes (0 h) and then 8 h, 16 h, and 24 h after adding approximately 1000 nematodes per plate. RNA was extracted using TRIzol (InvitrogenTM, Carlsbad, CA, USA) as previously described [38].

### 2.9. Real-Time PCR (RT-PCR) Analysis 

Levels of transcripts from *DdaCrz1* were measured using qPCR. RNAs extracted from strain 29 and its mutants were reversely transcribed into cDNA using a FastKing RT Kit with gDNase (Vazyme, Nanjing, China). The qPCR reactions were performed using SYBR Green Real-Time PCR Master Mix (Vazyme, Nanjing, China), and the *β-tubulin* gene was used as an internal standard. To calculate the relative transcriptional level of *DdaCrz1*, the 2^−ΔΔCt^ method was used [39], and these experiments were performed in triplicate.

### 2.10. Statistical Analysis

Experimental data were presented as mean ± SD. The *p*-values < 0.05 using Student’s *t*-test were considered statistically significant [36]. All statistical analyses were conducted using GraphPad Prism version 5.00 (GraphPad Software, San Diego, CA, USA).

## 3. Results

### 3.1. Identification of the Crz1 Gene of D. dactyloides 

**Figure 1:**
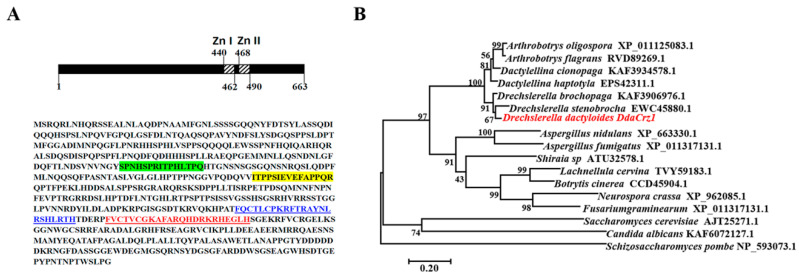
Characteristics of the DdaCrz1 protein and phylogenetic analysis. (**A**) The amino acid sequence of DdaCrz1. The residues in green and yellow boxes correspond to putative calcineurin-docking domains (CDD1 and CDD2). The underlined residues denoted using blue and red fonts indicate two C_2_H_2_-type zinc finger domains. (**B**) A maximum-likelihood phylogenetic tree showing 16 DdaCrz1 orthologs was built using MEGA7. The Crz1 protein encoded by *Schizosaccharomyces pombe* was used as the outgroup. Bootstrap values were indicated.

The gene (*DdaCrz1*) encoding the calcineurin-responsive Crz1 protein of *D. dactyloides* (CGMCC3.20198) produces a protein of 663 amino acids with a predicted molecular weight and pI of 73.22 kDa and 6.25, respectively. Two well-conserved zinc finger motifs (residues of 440–462 and 468–490) are present at the C-terminus (Figure 1A). Additionally, we identified two putative calcineurin-docking domains (Figure 1A). The sequence of DdaCrz1 is highly similar (77.73–91.47% identity with 100% coverage) to its orthologs encoded by other nematode-trapping fungi (Figure 1B), with the highest identity to those encoded by the constricting ring-forming fungi *D. brochopaga* (91.47%) and *D. stenobrocha* (82.28%). The sequence similarity to orthologs encoded by non-NTF in Ascomycetes such as *Aspergillus nidulans* (46.7% identity; 78% coverage) and yeast (significant similarity limited to the zinc finger motifs) was much lower.

### 3.2. DdaCrz1 Disruption and Complementation 

**Figure 2:**
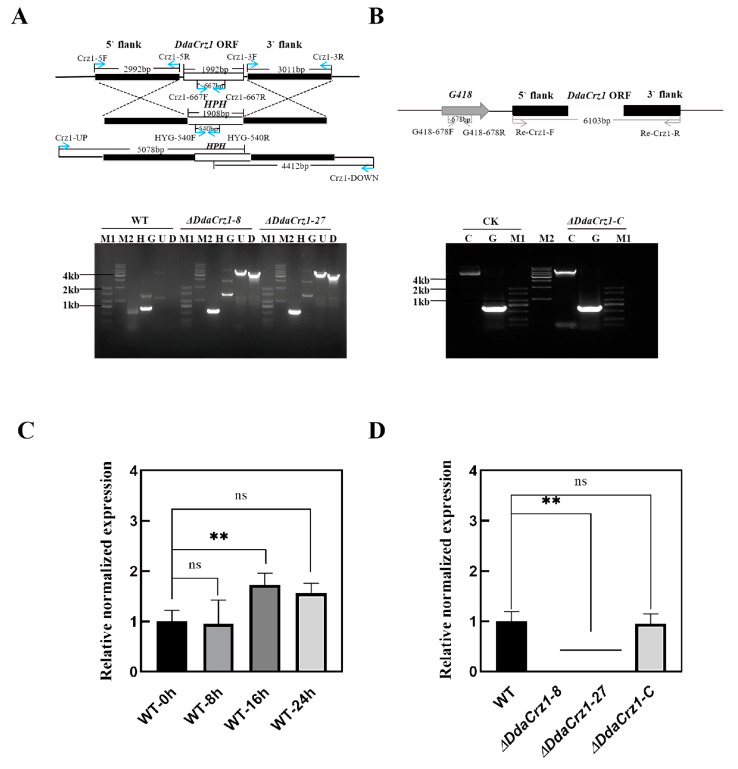
Strategies used for gene disruption and complementation and characterization of the resulting strains. (**A**) The strategy used for disrupting *DdaCrz1*. The primer locations and expected PCR product lengths are indicated. The gel image shows WT, the wild-type strain; M1, DNA marker D2000; M2, DNA marker 1 kb; H, part of the hygromycin resistance gene amplified using primers HYG-540F and HYG-540R, which was absent in WT and present in the mutants; G, part of the *DdaCrz1* open reading frame (ORF) amplified using primers Crz1-667F and Crz1-667R, which was absent in the mutants and present in WT; U, PCR products using primers Crz1-UP and HYG-540R; and D, PCR products using primers Crz1-DOWN and HYG-540F. (**B**) The strategy used to complement Δ*DdaCrz1*. The region amplified by PCR for complementation, primer locations, and expected PCR sizes are indicated. The gel image shows CK, the complementary plasmid used as a control; M1, DNA marker D2000; M2, DNA marker 1 kb; C, the complementary cassette detected using primers Re-Crz1-F and Re-Crz1-R; G, part of the G418 resistance gene amplified using primers G418-617F and G418-617R. (**C**,**D**) Quantitative real-time PCR of *DdaCrz1*. Two asterisks mean *p*-value < 0.01, two-tailed *t*-test, n = 3. ns means no significance found. (**C**) Expression patterns of *DdaCrz1* in the wild-type strain during the predation process. The samples analyzed include 0 h (without nematodes) and 8, 16, and 24 h after inducing with nematodes. (**D**) Expression levels of *DdaCrz1* in two *DdaCrz1* deletion mutants and one complemented *DdaCrz1* mutant without nematodes.

A homologous recombination-mediated strategy was applied to delete *DdaCrz1* (Figure 2A and Figure S1). Among 96 transformants obtained using ATMT, two deletion mutants (Δ*DdaCrz1-8* and Δ*DdaCrz1-27*) were selected (Figure S2) for subsequent analysis. Gene disruption was confirmed by PCR using genomic DNA extracted from these mutants (Figure 2A). A qRT-PCR analysis showed that *DdaCrz1* expression in strain 29 increased to 1.5-fold after introducing *C. elegans* for 16 h(Figure 2C), suggesting the involvement of *DdaCrz1* in the predatory process. Deletion of *DdaCrz1* was confirmed by the lack of gene expression (Figure 2D). The mutation was complemented by introducing a copy of the gene into Δ*DdaCrz1-27* via ATMT (Figure 2B), and one transformant (Δ*DdaCrz1-C*) was selected from 48 transformants (Figure S3). The expression level of *DdaCrz1* between strain 29 and the complemented mutant was similar (Figure 2D). 

### 3.3. Deletion of DdaCrz1 Decreased Vegetative Growth and Conidiation

**Figure 3:**
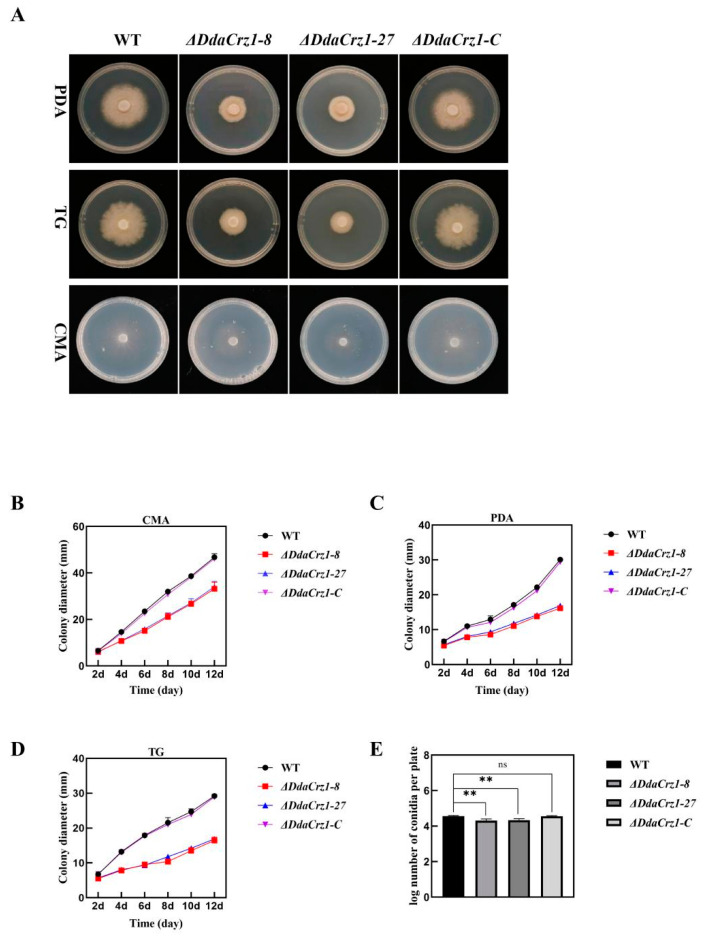
Colony growth and conidiation. (**A**) Colony morphology of strain 29 (WT), Δ*DdaCrz1*, and Δ*DdaCrz1-C* on potato dextrose agar (PDA), tryptone glucose (TG), and corn meal agar (CMA) media. (**B**–**D**) Colony growth on CMA, PDA, and TG media was measured every 2 days until the twelfth day. (**E**) Conidiation on CMA medium after 14 days of cultivation. Two asterisks mean *p*-value < 0.01, two-tailed *t*-test, n = 5. ns means no significance found.

Colony growth rates on CMA, PDA, and TG agar were similar between strain 29 and Δ*DdaCrz1-C*, but that of Δ*DdaCrz1* was significantly decreased. The colony diameters of strain 29 and Δ*DdaCrz1-C* were 46.7 ± 1.5 mm and 46.1 ± 0.8 mm, respectively, while two Δ*DdaCrz1* mutants were 33.2 ± 2.7 mm and 34.0 ± 2.3 mm in colony diameter after 12 days of incubation on CMA (Figure 3B and Table S2). The growth rates of the two Δ*DdaCrz1* mutants were 53.7% and 56.2% of that of strain 29 on PDA (Figure 3C and Table S3) and 56% and 58% on TG agar (Figure 3D and Table S4). The Δ*DdaCrz1* mutants formed sparse aerial hyphae compared with strain 29 and Δ*DdaCrz1-C* on all three media (Figure 3A). The number of conidia produced by the Δ*DdaCrz1* mutants was reduced (42% of that produced by strain 29 and Δ*DdaCrz1-C*) after 14 days of incubation on CMA supplemented with 2 g/L KH_2_PO_4_ (Figure 3E and Table S5).

### 3.4. Disruption of DdaCrz1 Decreased Cell Wall Integrity 

**Figure 4:**
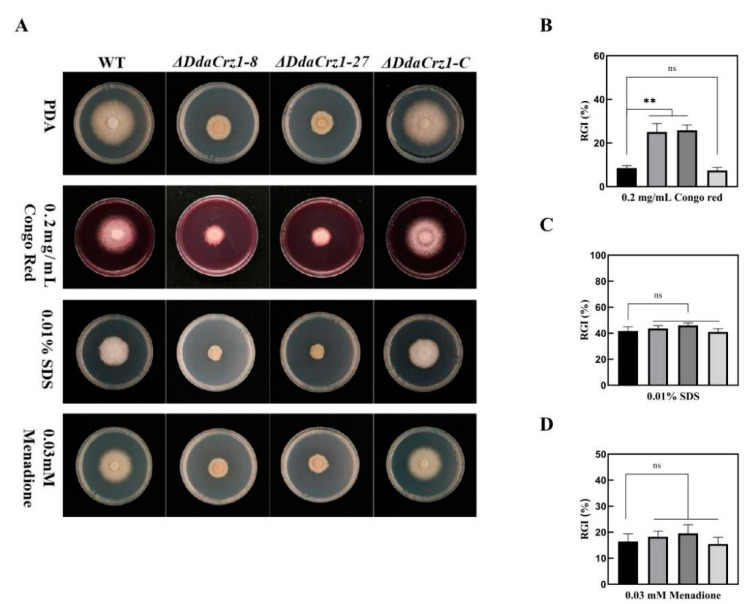
Colony morphology and growth in the presence of agents that cause cell-wall stress and oxidative stress. (**A**) Colony morphology of strain 29 (WT), Δ*DdaCrz1*, and Δ*DdaCrz1-C* on potato dextrose agar (PDA) containing 0.2 mg/ml Congo red, 0.01% SDS, and 0.03 mM Menadione. (**B**–**D**) Comparison of the growth inhibition of the WT, Δ*DdaCrz1*, and Δ*DdaCrz1-C* strains on PDA containing 0.2 mg/ml Congo red, 0.01% SDS, and 0.03 mM menadione. Two asterisks mean *p*-value < 0.01, two-tailed *t*-test, n = 5. ns means no significance found.

Cell wall permeability can be affected by Congo red (CR), a cell wall inhibitor that specifically binds to *β*-1,3-glucose, and SDS, a surfactant that removes lipids associated with the cell wall. CR and SDS were applied to test the involvement of *DdaCrz1* in maintaining cell wall integrity (CWI). The two Δ*DdaCrz1* mutants were more sensitive to CR (Figure 4A,B and Table S6), but not to SDS (Figure 4A,C and Table S6), in comparison with strain 29 and Δ*DdaCrz1-C* on PDA containing 0.2 mg/mL CR or 0.01% SDS, suggesting that *DdaCrz1* may regulate the expression of *β*-1,3-glucose synthase genes but is not involved in regulating lipid metabolism. 

### 3.5. Involvement of DdaCrz1 in Responding to Osmotic and Metal Ion Stresses

**Figure 5:**
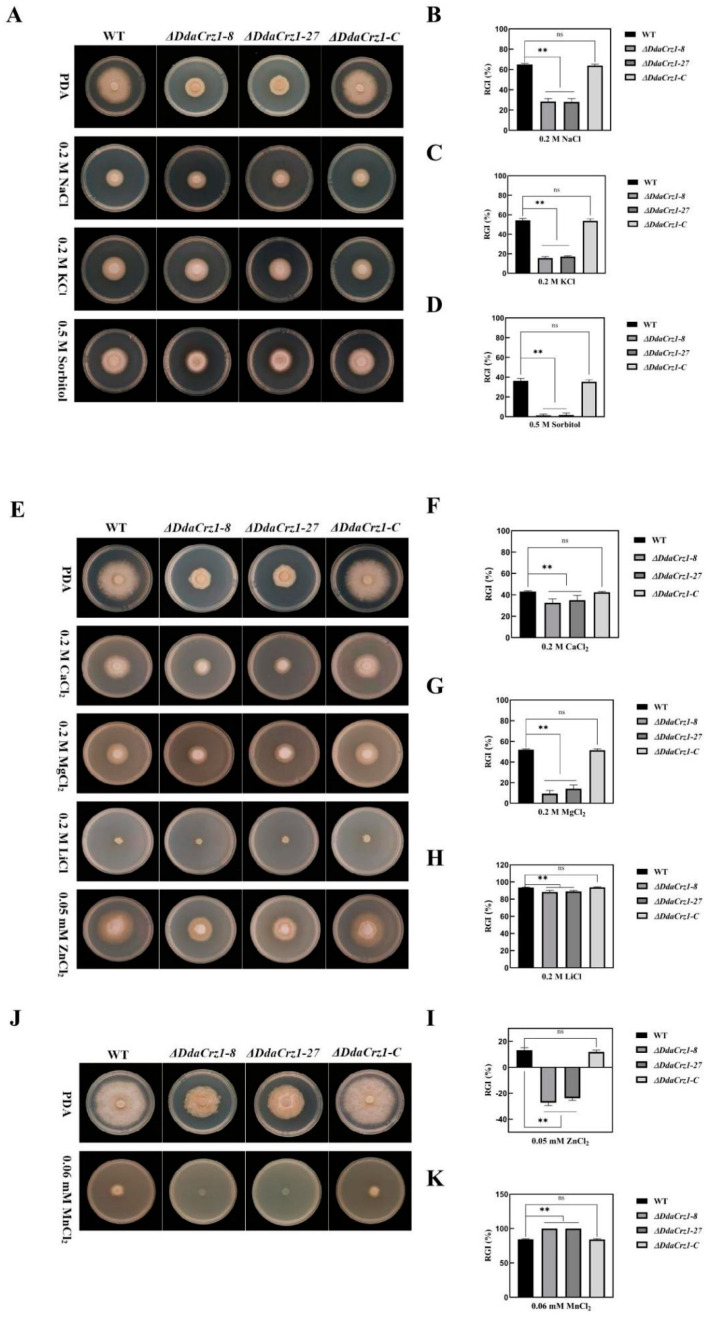
Colony morphology and growth under osmotic stresses and metal cation stresses. (**A**) Colony morphology of strain 29 (WT), Δ*DdaCrz1*, and Δ*DdaCrz1-C* on potato dextrose agar (PDA) containing 0.2 M NaCl, 0.2 M KCl, and 0.5 M sorbitol. (**B**–**D**) Relative growth inhibition rates of the WT, Δ*DdaCrz1*, and Δ*DdaCrz1-C* strains on PDA containing 0.2 M NaCl, 0.2 M KCl, and 0.5 M sorbitol. (**E**) Colony morphology of the WT, Δ*DdaCrz1*, and Δ*DdaCrz1-C* strains on potato dextrose agar (PDA) containing 0.2 M CaCl_2_, 0.2 M MgCl_2_, 0.2 M LiCl, and 0.05 mM ZnCl_2_. (**F**–**I**) Relative growth inhibition rates of the WT, Δ*DdaCrz1*, and Δ*DdaCrz1-C* strains on PDA containing 0.2 M CaCl_2_, 0.2 M MgCl_2_, 0.2 M LiCl, and 0.05 mM ZnCl_2_. (**J**) Colony morphology of the WT, Δ*DdaCrz1*, and Δ*DdaCrz1-C* strains on PDA containing 0.06 mM MnCl_2_. (**K**) Relative inhibition rates of the WT, Δ*DdaCrz1*, and Δ*DdaCrz1-C* strains on PDA medium containing 0.06 mM MnCl_2_. Two asterisks mean *p*-value < 0.01, two-tailed *t*-test, n = 5. ns means no significance found.

The Δ*DdaCrz1* mutants exhibited increased resistance to osmotic stresses on PDA containing 0.2 M NaCl, 0.2 M KCl, and 0.5 M sorbitol (Figure 5A–D; Table S7). However, strain 29, Δ*DdaCrz1-C*, and Δ*DdaCrz1* mutants showed similar susceptibility to 0.03 mM menadione (Figure 4A,D and Table S8). Δ*DdaCrz1* exhibited increased tolerance to Ca^2+^, Mg^2+^, Li^+^, and Zn^2+^ (Figure 5E–I and Table S9) but could not grow under Mn^2+^ stress (Figure 5J,K and Table S10). Crz1 in yeast has been documented to have high tolerance to Ca^2+^ and Mn^2+^ via the calcineurin-dependent induction of *PMC1* and *PMR1*, respectively [10], indicating that *DdaCrz1* may control the expression of the *D. dactyloides* gene homologous to *PMR1.*

### 3.6. Trap Formation and Ring Cell Inflation Affected by the Loss of DdaCrz1 

**Figure 6:**
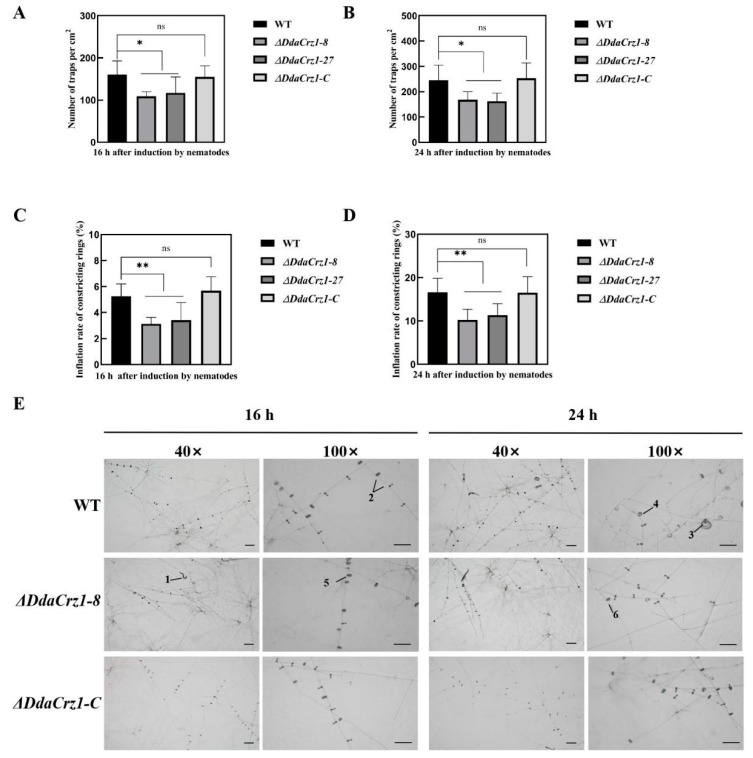
Trap formation and ring cell inflation by *D. dactyloides*. (**A**) The number of traps formed after 16 h of incubation with nematodes. (**B**) The number of traps formed after 24 h of incubation with nematodes. (**C**) Constricting ring inflation after 16 h incubation with nematodes. (**D**) Constricting ring inflation after 24 h of incubation with nematodes. One asterisk means 0.01 < *p*-value < 0.05 and two asterisks mean *p*-value < 0.01, two-tailed *t*-test, n = 5. ns means no significance found. (**E**) Trap formations by the WT, Δ*DdaCrz1-8*, and Δ*DdaCrz1-C* strains shown under both ×40 and ×100 magnification. 1, a free-living nematode; 2, uninflated constricting rings; 3, a trapped nematode; 4, 5, and 6, inflated constricting rings. Bar = 100 μm.

Capturing nematode is an essential part of the NTF lifestyle. The numbers of traps formed by the Δ*DdaCrz1* mutants after introducing *C. elegans* for 16 h and 24 h were 70% (16 h) and 58% (24 h), respectively (Figure 6A,B and Table S11)compared with those formed by strain 29. The trap number was restored in Δ*DdaCrz1-C*. The constricting ring inflation rates of the two Δ*DdaCrz1* mutants were lower than that of the WT at 16 h (3.3% vs. 5.3%) and 24 h (10.8% vs. 16.1%) after introducing *C. elegans* (Figure 6C,D and Table S12). There were no significant differences between Δ*DdaCrz1-C* and strain 29 (Figure 6). 

## 4. Discussion

Calcium ions, as second messengers, regulate a wide variety of cellular and developmental processes in eukaryotes. The calcium signaling pathway in fungi has been well characterized and is involved in growth, conidiation, thermo-tolerance, survival under ultraviolet light and oxidative stress, and sexual development [40]. For NTF, Ca^2+^/calmodulin-dependent protein kinases (CaMKs) and *AoFIG_1*, a member of the low-affinity Ca^2+^ transport system (LACS), have been shown to be involved in the growth, conidiation, stress response, and predatory ability of *A. oligospora* [24,25]. Disruption of *AoFIG_1* caused 90% reduction in trap formation, while deletion of *AoFIG_2* reduced the vegetative growth rate by up to 44% and led to the disappearance of traps and conidia [25]. Deletion of five orthologs of CaMKs in *A. oligospora* also reduced the ability to produce conidia and traps [24]. The role of *Crz1*, a gene under the control of calcineurin, in *D. dactyloides* was investigated in this study. The deletion of *DdaCrz1* not only impaired the growth, conidiation, and cell wall integrity but also interfered with the trap formation and ring cell inflation, supporting the involvement of the Ca^2+^ signaling in nematode trapping.

The Crz1 protein, a C_2_H_2_-type transcription factor controlled by Ca^2+^/calmodulin, is conserved among fungi and has been suggested to control the expression of glucan synthase, which regulates morphological and physiological traits. Consistent with previous reports on *B. cinerea*, *M. oryzae*, and *A. fumigatus* [17,18,19], the disruption of *DdaCrz1* led to significant defects in the growth, conidiation, and cell wall integrity of *D. dactyloides*. 

*Crz1* induces the expression of multiple genes responsive to ion stress and other stimulations in fungi [41,42]. The deletion of *DdaCrz1* made the mutant more susceptible to Mn^2+^, a trait observed in the *Crz1* mutants of *S. cerevisiae* *and A. fumigatus,* but not in the mutant of *B.* *cinerea* *and* *M. grisea* [10,17,18,43]. The Δ*DdaCrz1* mutants displayed increased resistance to Na^+^, K^+^, Mg^2+^, Li^+^, and Zn^2+^ stresses and the sensitivity to these cations of the *crz1* mutants was complex among different fungi [10,17,18,19,43], which suggested that the exact role of *Crz1* may be species-specific. Interestingly, the Δ*DdaCrz1* mutants exhibited reduced hypersensitivity to Ca^2+^ compared to strain 29, which is opposite to what has been observed in other fungi, such as *S. cerevisiae*, *A. fumigatus*, *M. oryzae*, and *B. cinerea*. The difference might be due to the predatory lifestyle of nematode-trapping fungi. 

The constricting ring (CR), a sophisticated trapping structure of NTF, mechanically captures nematodes by instantly inflating the ring cells when a vermiform nematode enters the ring cavity created by three ring cells. The mechanism underpinning ring constriction is poorly understood. Previous studies showed that applying inhibitors of calmodulin, calcium channels, and calcium pumps could decrease the percentages of inflated CRs in *D. dactyloides* [26]. Similar results were obtained when this pathway in *D. stenobrocha*, another constricting ring-forming fungus related to *D. dactyloides*, was disrupted. Chelation of Ca^2+^ using EGTA and the inhibition of calmodulin and calcium channels using trifluoperazine and ruthenium red/LaCl_3_ (two inhibitors of calcium channels) reduced the CR inflation rates of *D. stenobrocha* in a dosage-dependent manner, which indicated that Ca^2+^ was involved in regulating the inflation process (work in progress). The effect of *DdaCrz1* on the formation and inflation of constricting rings appeared to be significant (*p* < 0.05), but we cannot rule out the possibility that the reduced formation and inflation of constricting rings was simply due to the slower growth rate of the Δ*DdaCrz1* mutants. 

## Data Availability

Not applicable.

## Supplementary Materials

The following supporting information can be downloaded at: https://www.mdpi.com/article/10.3390/jof8070750/s1, Table S1: The primers used in this study, Figure S1: The PCR products corresponding to the *DdaCrz1* 5′ and 3′ flanking regions, Figure S2: PCR analysis of 96 ATMT transformants, Figure S3: PCR analysis of 48 ATMT transformants, Table S2: Colony diameter (mm) of the WT and mutants strains on CMA, Table S3: Colony diameter (mm) of the WT and mutant strains on PDA, Table S4: Colony diameter (mm) of the WT and mutant strains on TG, Table S5: Conidia numbers of the WT and mutant strains, Table S6: Colony diameter (mm) of the WT and mutant strains under cell wall stress, Table S7: Colony diameter (mm) of the WT and mutant strains under osmotic pressure, Table S8: Colony diameter (mm) of the WT and mutant strains under oxidative stress, Table S9: Colony diameter (mm) of the WT and mutant strains under metal cation pressure, Table S10: Colony diameter (mm) of the WT and mutant strains under metal cation pressure, Table S11: Constricting ring number of *D. dactyloides* after induction by nematodes, Table S12: Constricting ring inflation number of *D. dactyloides* after induction by nematodes.
