# Supplementary material for: DdaCrz1, a C2H2-Type Transcription Factor, Regulates Growth, Conidiation, and Stress Resistance in the Nematode-Trapping Fungus Drechslerella dactyloides
Source: J Fungi (Basel). 2022 Jul 20;8(7):750. doi: 10.3390/jof8070750 (PMC9322116; doi:10.3390/jof8070750)
Supplement: Supplementary file 1 [file jof-08-00750-s001.zip › jof-1759188-supplementary.pdf]

Supplementary materials

Table S1. Primers used in this thesis.

**Hygromycin resistance gene**	
HYG-540F	TTGCAAGACCTGCCTGAAACCGAACTGCCC
HYG-540R	AACCAAGCTCTGATAGAGTTGGTCAAGACC
**Geneticin resistance gene**	
G418-678F	GGCTATGACTGGGCACAACA
G418-678R	GATACCGTAAAGCACGAGGAA
DdaCrz1	
Crz1-5F	AACGACGGCCAGTGAATTCGAGCTCGGTACCCCCGTTGCAGATTAGTATGTCCAGTCT
Crz1-5R	GGGCCCATCGATGATCAGGCCTCGAGTTCGAGCGTTGGTGTCTTCGTGTTTCAT
Crz1-3F	CTAGTGCGCGATCGCGGCCGGCCGGCGCGCCGAGAGAGTGAGGCTTGGTGTTCGTCAG
Crz1-3R	GCTTGCATGCCTGCAGGTCGACATTAATTAAGACTGTATGTCCGGTACGTTATGGACG
Crz1-667F	TTCTTAGGTGGCCGGTGTTC
Crz1-667R	TCTGACATTTCACCCCAGCC
Crz1-UP	CGCATCGCTCGTCGTAATGATG
Crz1-DOWN	GTGAAGACCGGCTCGTCGTTCG
Re-Crz1-F	CCCCGAAGGTGAATACATGAACCAGTC
Re-Crz1-R	CGCAAGAGAATAATTCAAAGTCTGGTG
**RT-PCR**	
Tublin-RT-F	CAAGGTCAGCATGAAGGAG
Tublin-RT-R	CGATAATGAGGAGGAAGGT
Crz1-RT-F	CCTTGCCGCAGACGGTGCAG
Crz1-RT-R	CGAAGTCCCCACCCGTGGGC

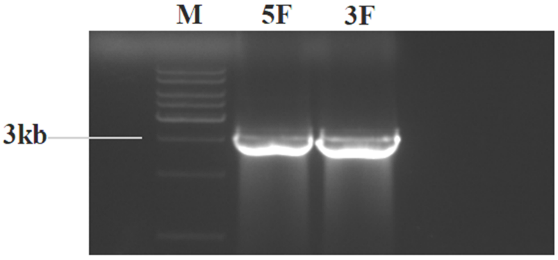

Figure S1. The PCR product lengths of *DdaCrz1* 5′ flanking and 3′ flanking. In the gel image, M, DNA marker 1 kb; 5F, 5′ flanking of *DdaCrz1* ORF; 3F, 3′ flanking of *DdaCrz1* ORF.

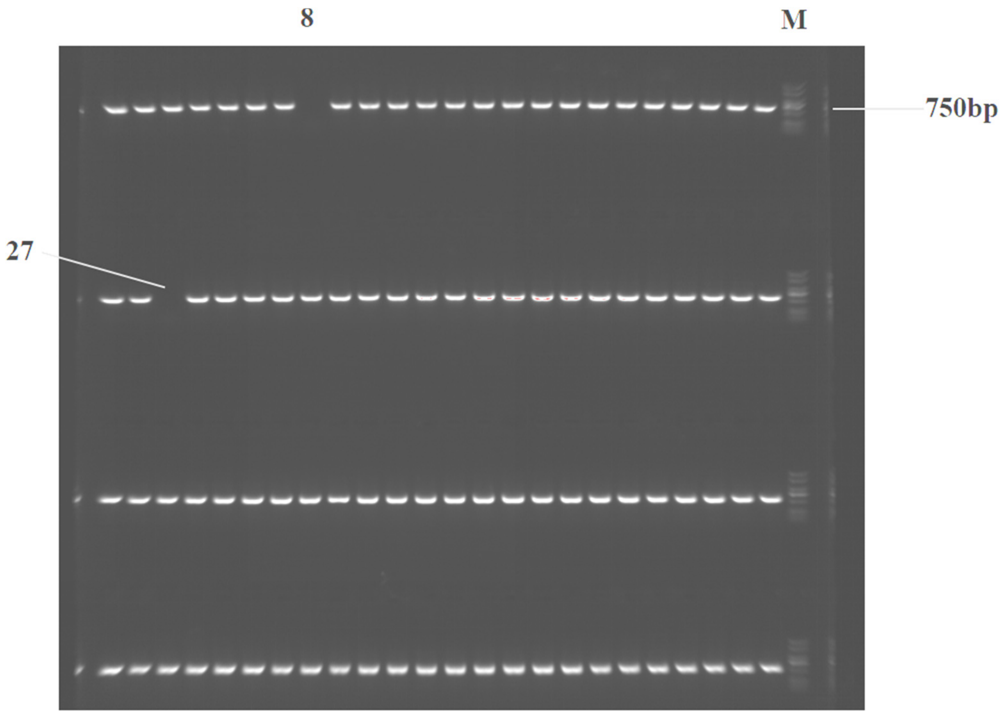

Figure S2. PCR verification for 96 ATMT transformants. In the gel image, 8, positive transformant *ΔDdaCrz1-8*; 27, positive transformant *ΔDdaCrz1-27*; M, DNA marker D2000.

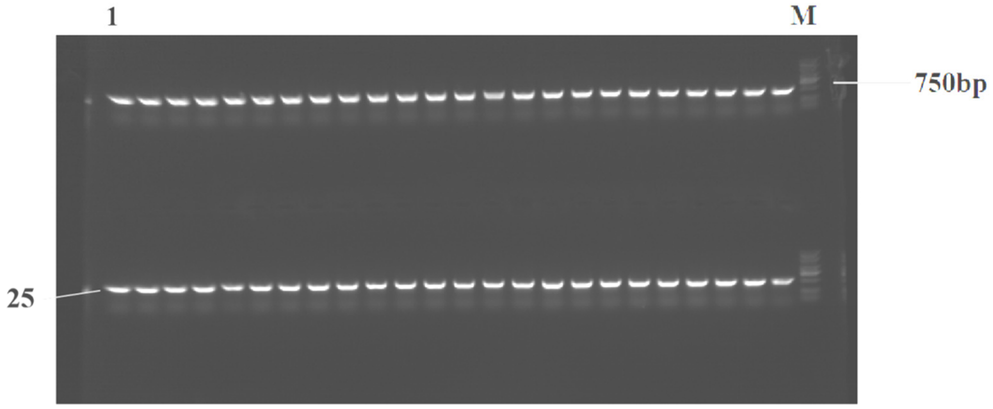

Figure S3. PCR verification for 48 ATMT transformants. In the gel image, M, DNA marker D2000, each lane means partial sequence of G418.

Table S2. Growth rate (colony diameter (mm)) of wild type (WT) strain and the mutants on CMA medium.

Strain	Rep1	Rep2	Rep3	Rep4	Rep5	Average	S.D.
**2-day-cultivation**							
WT	6.5	6.7	6.4	6.6	6.5	6.54	0.114
ΔDdaCrz1-8	6	5.9	6.1	6.2	6.5	6.14	0.230
ΔDdaCrz1-27	5.7	5.7	6	6.1	6.3	5.96	0.261
ΔDdaCrz1-C	6.3	6.4	6.6	6.8	6.2	6.46	0.241
**4-day-cultivation**							
WT	15	14.3	14.5	14.6	14.7	14.62	0.259
ΔDdaCrz1-8	10.1	11.7	10.8	10.6	10.5	10.74	0.594
ΔDdaCrz1-27	10.6	11.1	10.8	11.5	10.4	10.88	0.432
ΔDdaCrz1-C	13.6	13.5	13.7	14.5	14.2	13.9	0.430
**6-day-cultivation**							
WT	23.6	23.9	23.5	22.8	23.5	23.46	0.404
ΔDdaCrz1-8	13.8	16.2	14.1	14.7	16.5	15.06	1.226
ΔDdaCrz1-27	14.7	15.5	16.1	16.8	16.3	15.88	0.807
ΔDdaCrz1-C	22.3	21.9	22.8	23.5	22.1	22.52	0.642
**8-day-cultivation**							
WT	32	32.5	32.5	31	31.9	31.98	0.614
ΔDdaCrz1-8	19.6	21	20	21.3	23.4	21.06	1.483
ΔDdaCrz1-27	19.6	20.9	22.1	22.7	22.2	21.5	1.251
ΔDdaCrz1-C	31.8	29.5	30.2	31.9	30.3	30.74	1.060
**10-day-cultivation**							
WT	39.1	39.3	38.6	37.5	38.7	38.64	0.699
ΔDdaCrz1-8	26.1	26.9	25.4	26.4	28.6	26.68	1.203
ΔDdaCrz1-27	25.3	25.1	28.3	28.4	28.5	27.12	1.756
ΔDdaCrz1-C	38.6	37.5	37.5	38.6	38.3	38.1	0.561
**12-day-cultivation**							
WT	45.8	47.3	47.9	44.5	48	46.7	1.512
ΔDdaCrz1-8	29.6	31.4	34	34.2	36.7	33.18	2.743
ΔDdaCrz1-27	30.9	33.2	33.4	35.7	36.9	34.02	2.340
ΔDdaCrz1-C	46.9	46.2	44.8	45.8	46.7	46.08	0.834

Table S3. Growth rate (colony diameter (mm)) of wild type (WT) strain and the mutants on PDA medium.

Strain	Rep1	Rep2	Rep3	Rep4	Rep5	Average	S.D.
**2-day-cultivation**							
WT	6.9	6.6	6.8	6.7	6.2	6.64	0.27
ΔDdaCrz1-8	5.4	5.4	5.5	5.1	5.6	5.4	0.187
ΔDdaCrz1-27	5.9	5.5	5.2	6	5.8	5.68	0.327
ΔDdaCrz1-C	6.7	6.8	6.9	6.3	6.1	6.56	0.344
**4-day-cultivation**							
WT	11.9	11.5	10.8	10	10.9	11.02	0.726
ΔDdaCrz1-8	7.9	7.6	7.3	8.1	8.2	7.82	0.370
ΔDdaCrz1-27	7.9	8.1	7.6	8.4	8.3	8.06	0.321
ΔDdaCrz1-C	10.3	10.7	10.8	10.6	10.8	10.64	0.207
**6-day-cultivation**							
WT	14.2	11.8	13.8	12.2	12.6	12.92	1.035
ΔDdaCrz1-8	8.5	8.4	8.2	8.9	8.9	8.58	0.311
ΔDdaCrz1-27	9.6	9.7	9.2	9.2	9.1	9.36	0.270
ΔDdaCrz1-C	11.9	12	12.5	11.4	12.6	12.08	0.487
**8-day-cultivation**							
WT	17.7	16.4	16.9	17.5	17.1	17.12	0.512
ΔDdaCrz1-8	10.6	10.3	11.3	11.5	11.5	11.04	0.496
ΔDdaCrz1-27	12.1	11.7	11.4	12.5	11.2	11.78	0.526
ΔDdaCrz1-C	16.5	15.7	16.7	15.9	16.4	16.24	0.422
**10-day-cultivation**							
WT	23.4	21.9	21.7	21.4	22.3	22.14	0.777
ΔDdaCrz1-8	13.2	13.8	14.4	14.6	12.9	13.78	0.736
ΔDdaCrz1-27	14.2	13.5	13.6	15.2	14.6	14.22	0.709
ΔDdaCrz1-C	19.8	20.5	22	21.8	21.6	21.14	0.948
**12-day-cultivation**							
WT	30.9	29.6	29.8	29.6	30.5	30.08	0.589
ΔDdaCrz1-8	16.2	15.6	17.1	16.5	15.4	16.16	0.688
ΔDdaCrz1-27	17.3	16.8	16.2	17.3	16.9	16.9	0.453
ΔDdaCrz1-C	28.5	29.4	29.7	29.2	30.1	29.38	0.597

Table S4. Growth rate (colony diameter (mm)) of wild type (WT) strain and the mutants on TG medium.

Strain	Rep1	Rep2	Rep3	Rep4	Rep5	Average	S.D.
**2-day-cultivation**							
WT	6.3	7	6.8	6.5	7.1	6.74	0.336
ΔDdaCrz1-8	5.7	5.9	5.4	5.2	5.4	5.52	0.277
ΔDdaCrz1-27	5.9	5.5	6	5.4	5.7	5.7	0.255
ΔDdaCrz1-C	6.5	6.8	7	6.7	6.9	6.78	0.192
**4-day-cultivation**							
WT	12.6	13.5	12.7	13.6	13.6	13.2	0.505
ΔDdaCrz1-8	7.9	7.9	7.5	8.1	7.9	7.86	0.219
ΔDdaCrz1-27	7.9	7.9	7.9	8	8.4	8.02	0.217
ΔDdaCrz1-C	12.5	13.4	12.8	13.2	13.4	13.06	0.397
**6-day-cultivation**							
WT	17.1	17.5	18.5	18.2	18.2	17.9	0.579
ΔDdaCrz1-8	8.8	10.1	9.6	10	9	9.5	0.583
ΔDdaCrz1-27	9.2	8.7	9.4	10.1	10.1	9.5	0.604
ΔDdaCrz1-C	17.2	17.3	18	17.9	18.3	17.74	0.472
**8-day-cultivation**							
WT	21.6	20.1	20	23.3	22.7	21.54	1.491
ΔDdaCrz1-8	9.6	10.9	10	10.3	11.1	10.38	0.622
ΔDdaCrz1-27	10.1	10.1	10.9	11.4	11.2	10.74	0.611
ΔDdaCrz1-C	20.2	20.6	21.2	21.6	21.5	21.02	0.602
**10-day-cultivation**							
WT	24.6	23.4	24.9	25	25.7	24.72	0.841
ΔDdaCrz1-8	12.5	13.2	13.1	14.7	14.1	13.52	0.873
ΔDdaCrz1-27	12.6	13.4	14.8	13.7	14.5	13.8	0.880
ΔDdaCrz1-C	22.4	23.4	24.2	24.8	24.7	23.9	1.005
**12-day-cultivation**							
WT	29.8	29	28.5	29.2	29.5	29.2	0.495
ΔDdaCrz1-8	15	16.7	16.2	17.5	16.8	16.44	0.929
ΔDdaCrz1-27	16.3	17.5	17.5	16.5	17.5	17.06	0.607
ΔDdaCrz1-C	28.1	29	29.3	29.5	28.8	28.94	0.541

Table S5. Conidiation (conidia numbers) of wild type (WT) strain and the mutants.

Strain	Rep1	Rep2	Rep3	Rep4	Rep5	Average	S.D.
WT	39250	39000	33000	33500	38250	36600	3085.247
ΔDdaCrz1-8	17000	26000	19000	25250	16750	20800	4497.916
ΔDdaCrz1-27	22750	21000	25500	16250	24500	22000	3644.345
ΔDdaCrz1-C	36250	34500	38750	32000	38000	35900	2730.613

Table S6. Growth rate (colony diameter (mm)) of wild type (WT) strain and the mutants under cell wall stress.

Strain	Rep1	Rep2	Rep3	Rep4	Rep5	Average	S.D.
**14-day-cultivation PDA**							
WT	34.7	34.8	35.2	36.1	35.8	35.32	0.614
ΔDdaCrz1-8	16.2	16.8	18.2	17.1	16.9	17.04	0.730
ΔDdaCrz1-27	17.1	16.9	18	18.1	16.8	17.38	0.622
ΔDdaCrz1-C	35.2	35.6	34.9	34.3	35.5	35.1	0.524
**PDA containing 0.2 mg/mL Congo red**							
WT	32.7	33.1	32.3	33.1	32.5	32.74	0.358
ΔDdaCrz1-8	13.5	14.5	13.7	13.9	14.5	14.02	0.460
ΔDdaCrz1-27	14.5	14.5	13.9	14.1	13.9	14.18	0.303
ΔDdaCrz1-C	33.1	32.8	33.4	32.7	32.3	32.86	0.416
**PDA containing 0.01% SDS**							
WT	21.2	22.5	22.8	24	22.9	22.68	1.003
ΔDdaCrz1-8	11.8	11.4	11.7	11.9	12.1	11.78	0.259
ΔDdaCrz1-27	11.4	11.5	11.7	11.8	12.0	11.68	0.239
ΔDdaCrz1-C	22.6	21.8	22.5	23.7	23.1	22.74	0.709

Table S7. Growth rate (colony diameter (mm)) of wild type (WT) strain and the mutants under osmotic pressure.

Strain	Rep1	Rep2	Rep3	Rep4	Rep5	Average	S.D.
**14-day-cultivation PDA**							
WT	38.3	39.1	37.8	38.0	38.6	38.36	0.513
ΔDdaCrz1-8	22.9	22.3	22.9	22.8	22.3	22.64	0.313
ΔDdaCrz1-27	21.9	22.6	22.5	22.4	22.5	22.38	0.277
ΔDdaCrz1-C	38.5	37.7	38.4	38.5	38.2	38.26	0.336
**PDA containing 0.2 M NaCl**							
WT	17.4	16.9	16.9	16.3	16.5	16.8	0.424
ΔDdaCrz1-8	16.9	17.1	18.1	17.5	17.2	17.36	0.467
ΔDdaCrz1-27	18.2	17.2	17.1	18.3	17.2	17.6	0.596
ΔDdaCrz1-C	16.9	16.8	17.6	17.1	16.5	16.98	0.409
**PDA containing 0.2 M KCl**							
WT	20.8	19.2	20.6	20.9	20.2	20.34	0.691
ΔDdaCrz1-8	19.2	19.6	19.8	19.5	19.8	19.58	0.249
ΔDdaCrz1-27	19.4	19.3	19.5	19.6	19.7	19.5	0.158
ΔDdaCrz1-C	20.2	21.2	19.6	19.8	20.9	20.34	0.691
**PDA containing 0.5 M Sorbitol**							
WT	25.1	26.1	26.9	27.0	26.8	26.38	0.798
ΔDdaCrz1-8	22.1	22.2	22.4	21.9	21.8	22.08	0.239
ΔDdaCrz1-27	21.7	22.2	22.6	22.3	22.1	22.18	0.327
ΔDdaCrz1-C	26.2	27.1	25.9	27.2	25.8	26.44	0.666

Table S8. Growth rate (colony diameter (mm)) of wild type (WT) strain and the mutants under oxidative stress.

Strain	Rep1	Rep2	Rep3	Rep4	Rep5	Average	S.D.
**14-day-cultivation PDA**							
WT	34.7	34.8	35.2	36.1	35.8	35.32	0.614
ΔDdaCrz1-8	16.2	16.8	18.2	17.1	16.9	17.04	0.730
ΔDdaCrz1-27	17.1	16.9	18	18.1	16.8	17.38	0.622
ΔDdaCrz1-C	35.2	35.6	34.9	34.3	35.5	35.1	0.524
**PDA containing 0.03 mM Menadione**							
WT	31.8	29.8	30.6	29.7	29.8	30.34	0.893
ΔDdaCrz1-8	15.1	14.5	15.1	14.7	14.8	14.84	0.261
ΔDdaCrz1-27	14.3	15.1	15.4	14.9	15.1	14.96	0.410
ΔDdaCrz1-C	31.5	30.8	29.7	30.5	29.7	30.44	0.767

Table S9. Growth rate (colony diameter (mm)) of wild type (WT) strain and the mutants under metal cation pressure.

Strain	Rep1	Rep2	Rep3	Rep4	Rep5	Average	S.D.
**14-day-cultivation PDA**							
WT	35.7	36.4	36.8	36.7	37.0	36.52	0.507
ΔDdaCrz1-8	17.8	19.0	20.0	19.9	19.5	19.24	0.896
ΔDdaCrz1-27	18.9	19.6	19.8	19.6	19.4	19.46	0.344
ΔDdaCrz1-C	35.6	34.9	36.7	36.8	36.9	36.18	0.887
**PDA containing 0.2 M $CaCl_2$**							
WT	22.9	22.8	22.6	23.2	23.2	22.94	0.261
ΔDdaCrz1-8	15.0	14.2	13.9	14.9	15.0	14.6	0.515
ΔDdaCrz1-27	15.1	14.7	14.2	13.4	14.5	14.38	0.638
ΔDdaCrz1-C	22.7	22.8	22.9	23.1	23.2	22.94	0.207
**PDA containing 0.2 M $MgCl_2$**							
WT	20.2	20.3	19.8	20.0	20.1	20.08	0.192
ΔDdaCrz1-8	17.8	18.6	17.6	18.0	17.5	17.9	0.436
ΔDdaCrz1-27	16.8	17.4	18.2	17.5	17.2	17.42	0.512
ΔDdaCrz1-C	19.6	20.2	19.8	20.5	20.6	20.14	0.434
**PDA containing 0.2 M LiCl**							
WT	6.9	7.2	6.9	7.0	7.2	7.04	0.152
ΔDdaCrz1-8	6.3	6.8	6.9	6.8	6.5	6.66	0.251
ΔDdaCrz1-27	6.6	6.3	6.8	6.6	6.7	6.6	0.187
ΔDdaCrz1-C	7.0	7.3	6.8	6.9	6.8	6.96	0.207
**PDA containing 0.05 mM $ZnCl_2$**							
WT	31.8	32	32.2	33.3	32.4	32.34	0.581
ΔDdaCrz1-8	23.2	22.7	23.3	22.9	23.5	23.12	0.319
ΔDdaCrz1-27	23.0	23.2	22.7	22.6	23.0	22.9	0.245
ΔDdaCrz1-C	32.0	32.2	32.8	33.1	32.4	32.5	0.447

Table S10. Growth rate (colony diameter (mm)) of wild type (WT) strain and the mutants under metal cation pressure.

Strain	Rep1	Rep2	Rep3	Rep4	Rep5	Average	S.D.
**25-day-cultivation PDA**							
WT	50.4	50.5	50.3	50.3	50.2	50.34	0.114
ΔDdaCrz1-8	32	31.4	31.2	31.5	31.7	31.56	0.305
ΔDdaCrz1-27	31.3	31.4	31.4	31.5	31.8	31.48	0.192
ΔDdaCrz1-C	49.9	50.5	50.3	50.9	49.7	50.26	0.477
**PDA containing 0.06 mM $MnCl_2$**							
WT	12.5	11.8	12	11.7	12.3	12.06	0.336
ΔDdaCrz1-8	5.0	5.0	5.0	5.0	5.0	5.0	0
ΔDdaCrz1-27	5.0	5.0	5.0	5.0	5.0	5.0	0
ΔDdaCrz1-C	12.6	11.9	11.5	12.3	12.4	12.14	0.439

Table S11. Trap number of *D. dactyloides* after induction by nematodes.

Strain	Rep1	Rep2	Rep3	Rep4	Rep5	Average	S.D.
**16 h after induction by nematodes**							
WT	152	116	197	188	147	160	32.871
ΔDdaCrz1-8	103	98	115	124	104	108.8	10.521
ΔDdaCrz1-27	169	143	89	105	79	117	37.921
ΔDdaCrz1-C	130	124	178	165	177	154.8	25.975
**24 h after induction by nematodes**							
WT	293	198	267	168	301	245.4	59.290
ΔDdaCrz1-8	121	178	187	201	154	168.2	31.444
ΔDdaCrz1-27	169	186	145	116	195	162.2	32.089
ΔDdaCrz1-C	188	256	197	301	323	253	60.361

Table S12. Constricting ring inflation number of *D. dactyloides* after induction by nematodes.

Strain	Rep1	Rep2	Rep3	Rep4	Rep5	Average	S.D.
**16 h after induction by nematodes**							
WT	8	7	8	11	8	8.4	1.5166
ΔDdaCrz1-8	4	3	3	4	3	3.4	0.548
ΔDdaCrz1-27	5	6	4	3	2	4	1.581
ΔDdaCrz1-C	8	7	11	10	8	8.8	1.643
**24 h after induction by nematodes**							
WT	45	32	34	51	48	42	8.515
ΔDdaCrz1-8	15	13	24	22	17	18.2	4.658
ΔDdaCrz1-27	19	15	16	17	25	18.4	3.975
ΔDdaCrz1-C	35	39	41	36	58	41.8	9.365
